# Glycaemic Index of Maternal Dietary Carbohydrate Differentially Alters *Fto* and *Lep* Expression in Offspring in C57BL/6 Mice

**DOI:** 10.3390/nu10101342

**Published:** 2018-09-20

**Authors:** Theodora Sideratou, Fiona Atkinson, Grace J. Campbell, Peter Petocz, Kim S. Bell-Anderson, Jennie Brand-Miller

**Affiliations:** 1 School of Life and Environmental Sciences, University of Sydney, Sydney, NSW 2006, Australia; dorasider@hotmail.com; 2 School of Life and Environmental Sciences and Charles Perkins Centre, University of Sydney, Sydney, NSW 2006, Australia; fiona.atkinson@sydney.edu.au (F.A.), grace.campbell@sydney.edu.au (G.J.C.), jennie.brandmiller@sydney.edu.au (J.B.-M.); 3 Department of Statistics, Macquarie University, Sydney, NSW 2109, Australia; peter.petocz@mq.edu.au

**Keywords:** glycaemic index, obesity, gene expression, appetite, FTO, C57BL/6, pregnancy

## Abstract

Maternal diet and gestational hyperglycaemia have implications for offspring health. Leptin (*LEP*) and fat mass and obesity-associated (*FTO*) alleles are known to influence body fat mass in humans, potentially via effects on appetite. We hypothesized that expression of *Fto*, *Lep*, and other appetite-related genes (*Argp, Npy, Pomc, Cart, Lepr*) in the offspring of female mice are influenced by the glycaemic index (GI) of carbohydrates in the maternal diet. C57BL/6 mice were randomly assigned to low or high GI diets and mated with chow-fed males at eight weeks of age. Male pups were weaned at four weeks and randomly divided into two groups, one group following their mother’s diet (LL and HH), and one following the standard chow diet (LC and HC) to 20 weeks. *Fto* expression was 3.8-fold higher in the placenta of mothers fed the high GI diet (*p* = 0.0001) and 2.5-fold higher in the hypothalamus of 20-week old offspring fed the high GI (HH vs. LL, *p* < 0.0001). By contrast, leptin gene (*Lep*) expression in visceral adipose tissue was 4.4-fold higher in four-week old offspring of low GI mothers (LC vs. HC, *p* < 0.0001) and 3.3-fold higher in visceral adipose tissue of 20-week old animals (LL vs. HH, *p* < 0.0001). Plasma ghrelin and leptin levels, and hypothalamic appetite genes were also differentially regulated by maternal and offspring diet. These findings provide the first evidence in an animal model that maternal high GI dietary carbohydrates that are digested and absorbed faster may contribute to programming of appetite in offspring.

## 1. Introduction

Obesity and type 2 diabetes have increased globally over recent decades and appear at increasingly younger ages [1]. Maternal obesity and gestational diabetes are associated with higher birthweight and foetal adiposity [2]. Foetal metabolism is largely based on the principal energy substrate, glucose [3], which is transported across the placenta at the same glucose concentration as the maternal circulation. Maternal glucose levels that are in the high-normal range but below the diagnostic values of gestational diabetes are also associated with increased birth weight and high foetal insulin levels [4,5]. In normal pregnancy and those affected by gestational diabetes, dietary interventions that reduce postprandial glycaemia, including low glycaemic index (GI) diets, have been associated with improved pregnancy outcomes [6,7,8,9,10].

Despite the importance of the early life environment, genetic factors contribute markedly to the risk of obesity. The most compelling evidence of genetic involvement is the fat mass and obesity-associated *FTO* gene located within the chromosome 16q12.2 region, and expressed mainly in the hypothalamus [11,12,13]. *FTO* is not only statistically the strongest associated gene but also has the largest effect size on obesity risk [14], yet its physiological role and mechanism of action remain unclear. High expression in the hypothalamus, the regulatory centre of energy balance, suggests that *FTO* influences adiposity through its impact on appetite and energy homeostasis [15]. Other hormones produced in the hypothalamus play counter-regulatory roles in stimulating appetite (e.g., agouti-related peptide and neuropeptide Y) or promoting satiety (e.g., pro-opiomelanocortin cocaine, amphetamine regulated transcript, and leptin receptor), depending on the physiological state of the animal [16].

We hypothesised that the expression of *FTO* and other genes involved in regulating offspring body mass and appetite may be differentially influenced by the carbohydrate quality of maternal diets [17,18]. In particular, changes in food processing have corresponded to higher temperatures and pressures and degrees of starch gelatinization, thereby increasing the rate of digestion of starch in individual foods and overall glycaemic response [19,20]. Processing temperatures also influence the crystallization of amylose starch and the amount of retrograded or resistant starch. In turn, this changes the amount of starch available for small intestinal digestion and the amount that reaches the large bowel for microbial fermentation. The GI is a metric that compares the degree of postprandial glycaemia elicited by a single food to that of a reference food (usually glucose) with the same amount of available carbohydrate (either 25 or 50 g) using a standardized protocol [21]. Diets with a lower GI have been shown to influence the rate of weight loss [22], weight re-gain [23,24,25], maternal glucose tolerance [26,27], and glucose control in individuals with diabetes [28]. In animal studies, rapidly vs. slowly digested starch (high GI vs. low GI) diets result in increased body fat [29], impairments in glucose tolerance, and disrupted beta-cell function [29,30].

In the present study our aim was to compare markers of obesity risk in offspring of female mice fed diets based on rapidly or slowly digested starch, but otherwise identical nutrient composition. Specifically, using a mouse model, we compared the expression of *Fto* in placenta and hypothalamus, leptin (*Lep*) in adipose tissue and five appetite-associated genes in the hypothalamus: agouti-related peptide (*Agrp*), neuropeptide Y (*Npy*), pro-opiomelanocortin cocaine (*Pomc*), amphetamine-regulated transcript (*Cart*), and leptin receptor (*Lepr*) in specific tissues. Our study design allowed for observations of both early-life feeding and longer exposure to rapidly vs. slowly digested starch.

## 2. Materials and Methods

### 2.1. Animals and Diets

The study protocol was approved by the Animal Ethics Committee of the University of Sydney (Protocol number L02/8-2010/2/5355). The authors assert that all procedures contributing to this work comply with the ethical standards of the Australian code for the care and use of animals for scientific purposes (8th edition). Animals were provided by the Animal Resources Centre (ARC, Canning Vale, WA, Australia). Mice breeders were kept in indoor animal house facilities at 22 °C, where 2–6 same-sex mice were housed together in standard cages. Mice had ad libitum access to feed and water through a feeder tray within the lid of the cage. The light-dark cycle of the animal holding room was 12 h–12 h.

Two custom diets and a standard rodent chow were obtained commercially from Specialty Feeds (Glen Forrest, WA, Australia). The two custom diets had identical macronutrient composition and calculated energy density, with only the starch component being different (Supplementary Table S1). The starch was mechanically manipulated in order to obtain a ‘gel crisp’ high amylose starch for the low GI diet (30% amylopectin, 70% amylose) and a dextrinised high amylopectin starch for the high GI diet (90% amylopectin, 10% amylose). Both starches were extracted from conventionally-bred varieties of maize (corn, *Zea mays*). The diets were not irradiated and were enhanced with small amounts of AIN-93 vitamin premix that used wheat starch instead of sucrose or glucose as the carrier.

### 2.2. In Vitro GI Testing of the Diets

A standardised starch digestion assay [31] was performed to demonstrate differences in rate of digestion between the two diets. The enzyme solution was prepared by suspending porcine pancreatic α-amylase (150 U/mg, Sigma-Aldrich, St. Louis, MO, USA) in water with magnetic stirring for 10 min at 37 °C. The mixture was centrifuged (1500× *g* for 10 min) and amyloglucosidase (3260 U/mL, Megazyme International Ireland Ltd., Bray, County Wicklow, Ireland) was added. The diets were crushed into a fine powder and the amount corresponding to 100 mg starch (dry weight) was dispersed in 0.1 M sodium acetate buffer (pH 5.2) and freshly prepared enzyme solution. This mixture was incubated in a shaking water bath (160 strokes/min at 37 °C) and aliquots (0.1 mL) were taken at specified intervals (0, 20, 120, and 180 min) and mixed with 95% ethanol. Free glucose released was measured using the glucose oxidase-peroxidase reaction (Megazyme International Ireland Ltd., Bray, County Wicklow, Ireland). Rapidly and slowly available glucose were determined as measures of the rate at which glucose from each diet became available for absorption. Rapidly available glucose is defined as the glucose amount released within the first 20 mins of the start of incubation, and slowly available glucose as the amount released between 20 to 120 mins.

### 2.3. Animals

C57BL/6 female mice were obtained from ARC at 4 weeks of age and randomly assigned to one of the following diet groups: low GI diet (LGI), high GI diet (HGI) or standard chow (C). C57BL/6 male breeders were obtained from ARC at 3 weeks of age and consumed chow for 4 weeks prior to mating. Male pups from the LGI and HGI groups were weaned at the beginning of week 5 and divided into two subgroups, either following their mother’s diet (LL and HH, respectively) or a chow diet (LC and HC, respectively). Male pups of the C group were weaned at the same time and continued on chow (CC) and used as a reference group. A schematic representation of the study design is shown in Supplementary Figure S1.

### 2.4. Food Intake and Weight Monitoring

After weaning, pup weights were recorded weekly until 20 weeks of age upon which they were sacrificed. Three 24-h food intake observations were conducted at weeks 9, 12, and 16. Mice were placed in custom-made cages that contained an insert to allow collection of spilled food. The experiment was conducted according to cage grouping (*n* = 2–5 per cage group) to minimise stress of individual housing. Food intake measurements were recorded by weighing food pellets before and after 24 h. Food spilled into the bottom of the cage was subtracted.

### 2.5. Glucose and Insulin Tolerance Tests

Intraperitoneal glucose and insulin tolerance tests (GTT, ITT) were conducted at weeks 17 and 18, respectively. We used the intraperitoneal injection to assess glucose tolerance because it measures glucose tolerance *per se* without being influenced by differences in secretion of gut-derived hormones such as GIP and GLP-1 (as occurs with oral glucose tolerance tests). Mice were fasted for 4–6 hours prior to testing [32,33]. Basal blood glucose levels were determined using an Accu-Check Performa glucometer (Roche Diagnostics Australia Pty Ltd., Victoria Avenue, Castle Hill NSW, Australia) through a small incision on the tail. Mice were administered glucose (2 mg/g body weight of 50% *w*/*v* solution) or insulin (1 U/kg body weight of Actrapid^®^, Novo Nordisk Pharmaceuticals Baulkham Hills, Australia) via an intraperitoneal injection. Blood glucose levels were determined at 15, 30, 45, 60, 90, and 120 min after injection. The incremental area under the curve (iAUC) was calculated in accordance with the method recommended by Wolever et al. [34]. During the ITT, mice with low readings at any point were given a glucose injection and removed from the experiment. The rate of decline of glucose (slope) during the first 30 min after insulin injection was measured and interpreted as a measure of insulin sensitivity.

### 2.6. Mouse Sacrifice, Blood and Tissue Collection

At 20 weeks of age, 4–6 h fasted mice were sacrificed by intraperitoneal injection with Lethabarb (Virbac, Penrith, NSW, Australia). With a cardiac puncture, blood was collected into sterile, heparin-coated tubes and separated by centrifugation. Plasma was transferred to a fresh tube, snap frozen in liquid nitrogen and stored at −80 °C until analysed. Hypothalamus, brown adipose tissue (BAT), liver, visceral adipose tissue (VAT), subcutaneous adipose tissue (SAT), red muscle (RM) and white muscle (WM) tissues were collected, freeze-clamped in liquid nitrogen, and stored at −80 °C until analysed.

A sub-group of pregnant mice from the HGI and LGI groups was sacrificed at day 18–19 of gestation by lethal dose of isoflurane solution [35,36]. A small incision was made to access the peritoneum and the uterine horns located and placed in a Petri dish containing phosphate-buffered saline (PBS) solution. The amniotic sac was opened and each foetus separated from the placenta by a slicing incision on the umbilical cord and pulling the cord and the attached foetus away from the placenta. The placenta was dissected, freeze-clamped in liquid nitrogen and stored at −80 °C until analysed.

### 2.7. Quantitative Real-Time PCR (qRT-PCR)

Total RNA extraction was performed using either RNeasy Mini kit (hypothalamus and placenta) or RNeasy Lipid Tissue Mini Kit (BAT, liver, VAT, SAT, RM, WM) by Qiagen (Chadstone, VIC Australia) as per manufacturer’s instructions. The RNA purity and yield were determined using a NanoDrop (ND-1000) spectrophotometer (NanoDropTechnologies Inc., Wilmington, DE, USA) and by gel electrophoresis. Total RNA was reverse transcribed from 200 ng of RNA, using Superscript^®^ VILO™ cDNA Synthesis Kit (Life Technologies, Gaithersburg, MD, USA) according to the manufacturer’s instructions. The primer sequences of the two target genes (*Fto* and leptin) were designed using the ‘Primer3′ (version 0.4.0) web tool available at http://frodo.wi.mit.edu/primer3/ [37,38]. Primers were synthesised by Sigma-Genosys (Sigma-Aldrich, Castle Hill, NSW, Australia). The sequences of the oligonucleotides are shown in Supplementary Table S2. Real-time PCR was performed in an ABI 7500 instrument (Applied Biosystems, Foster City, CA, USA) using SYBR^®^ Green Master Mix (Applied Biosystems, Foster City, CA, USA) according to the manufacturer’s instruction. The qRT-PCR runs consisted of an intial 5 min 50 °C hold, and 40 cycles of 3 sec at 95 °C and 30 sec at 60 °C. Cycle threshold (Ct) values were normalised to 18S rRNA Ct value, which was used as the reference sequence. Gene expression was estimated using the Ct (2ΔΔct) method and is expressed as fold change compared to control, i.e., the chow-fed offspring of chow-fed mothers (CC) [39].

### 2.8. Plasma Analysis

Plasma ghrelin, leptin, adiponectin, and insulin concentrations were determined in samples collected at sacrifice by ELISA using a Multiplex Map Kit by Cardinal Bioresearch Pty Ltd. (Wollongong, NSW, Australia). Protease inhibitors were added during sample thawing.

### 2.9. Statistical Analysis

Data were analysed using SPSS Statistics version 19 (IBM, Chicago, IL, USA) and Prism 6.0 (Graph Pad Software, GA, USA). One-way repeated measures analysis of variance (ANOVA) was carried out to assess the difference between the studied variables among the groups, using repeated-measures analysis for variables measured longitudinally. Pairwise comparisons were carried out post hoc with a Bonferroni correction for multiple testing. Statistical significance was defined as *p* < 0.001 and marginal significance at *p* ≤ 0.05. A normal distribution was evident for most variables but in the case of plasma hormone levels, a large within-group variability was observed and significant deviation from normality. In these instances, Kruskal–Wallis and Mann-Whitney U non-parametric tests were used.

## 3. Results

### 3.1. Rate of Starch Digestion in Vitro

**Figure 1:**
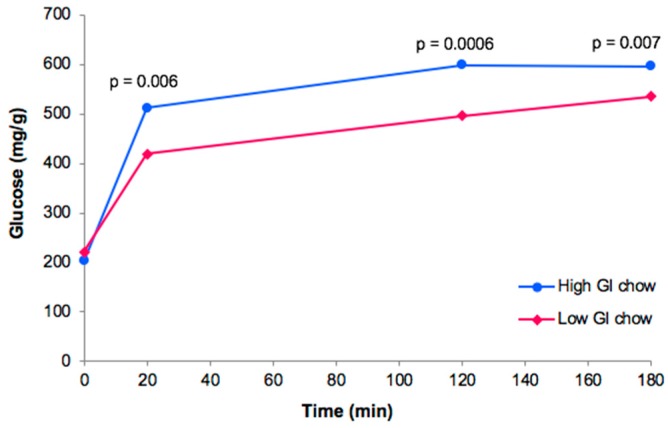
Rate of starch digestion for the high GI and the low GI diets. Rate of digestion was determined by the Englyst procedure [31]. Diets were tested in duplicate on three separate occasions.

Using the rate of digestion *in vitro* as a surrogate, we confirmed that the custom high GI diet contained more rapidly digestible starch than the low GI diet. At 20 min, 56% more glucose had been released by the high GI diet compared with the low GI diet (*p* = 0.006, Figure 1). Similarly, at 120 min 44% more glucose had been released (*p* = 0.0006, Figure 1).

### 3.2. Food Intake and Body Weight

**Figure 2:**
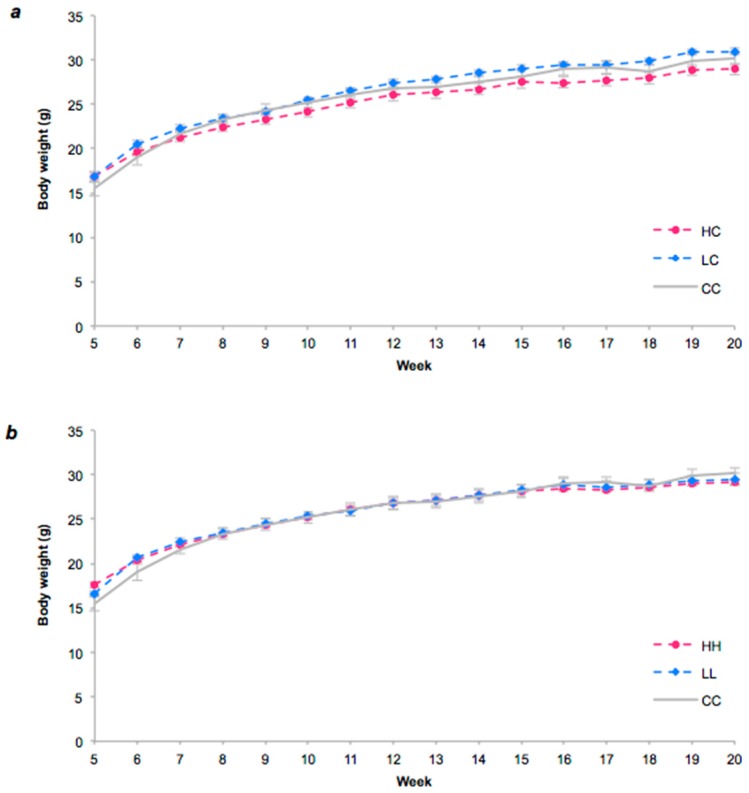
Body weight was measured weekly from weaning in offspring fed five different diets. Figure 2(**a**). CC (*n* = 13) denotes the group whose mothers received chow (C) throughout pregnancy and lactation and continued on chow from weaning to 20 weeks. HC (*n* = 13) denotes offspring whose mothers received the high GI diet (H) and were switched to chow from weaning onwards. LC (*n* = 14) denotes offspring whose mothers received the low GI diet (L) but were switched to chow from weaning onwards. Figure 2(**b**). HH (*n* = 15) denotes offspring whose mothers received the high GI diet and remained on the high GI from weaning onwards. LL (*n* = 10) denotes offspring whose mothers received the low GI diet and continued on the low GI diet from weaing onwards. Data are expressed as the Mean ± SEM. There were no significant differences except at week 16 in HC vs. LC.

There were no significant differences in body weight of the pups from weaning to 12 weeks of age (Figure 2). At weeks 16 and 19, LC offspring showed a marginally higher body weight than HC offspring (*p* = 0.005).

### 3.3. Glucose and Insulin Tolerance Tests

**Figure 3:**
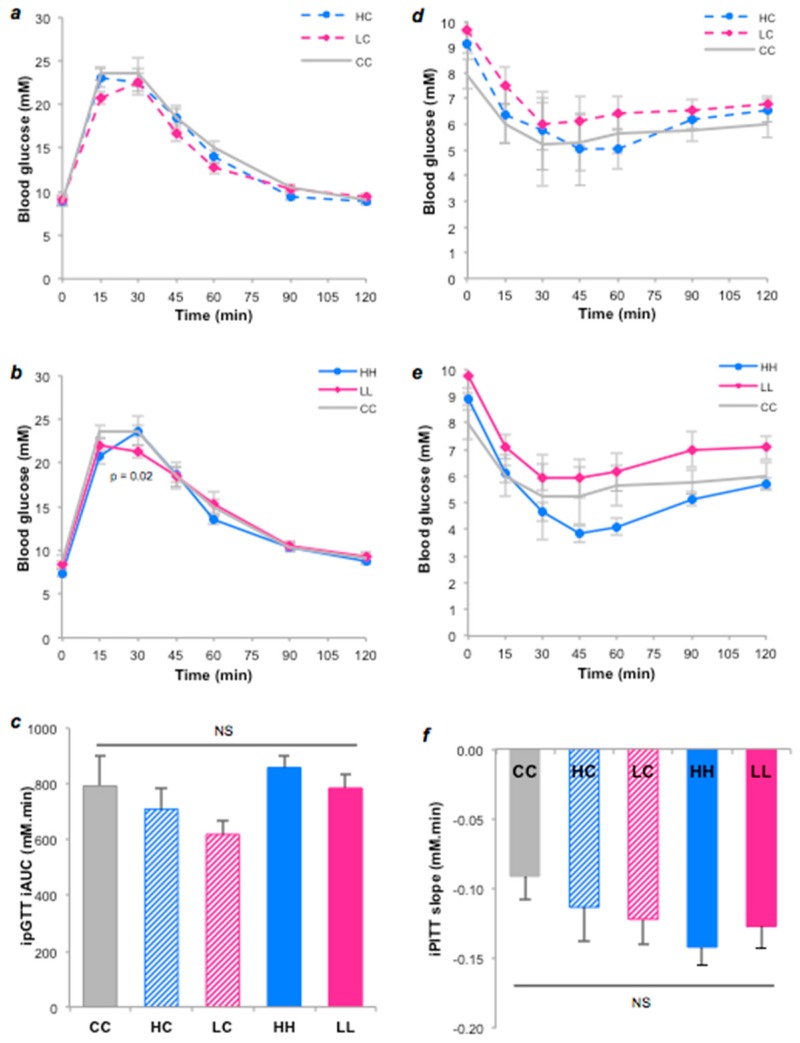
Metabolic responses to the intraperitoneal glucose tolerance and insulin tolerance tests in 17–18 week old offpring fed 5 different diets. Blood glucose responses (**a, b**) and iAUC (**c**) after glucose injection. Blood glucose responses (**d**, **e**) and the slope of blood glucose change in first 30 mins (**f**) in response to intraperitoneal insulin injection. Data represent the Mean ± SEM. NS = not significant. For glucose tolerance tests: CC *n* = 8, HC *n* = 10, HH *n* = 10, LC *n* = 13, LL *n* = 10. For insulin tolerance tests: CC *n* = 7, HC *n* = 7, HH *n* = 12, LC *n* = 7, LL *n* = 10.

Overall, there were no significant differences in acute blood glucose responses (iAUC) between the groups, implying similar glucose tolerance (Figure 3a,b,c). However, peak glucose at 15 min was marginally higher in HC vs. LC (*p* < 0.05), while glucose concentration at 30 min was marginally higher in HH vs. LL (*p* = 0.02), implying slower glucose clearance in those exposed to high GI starch. However, there were no statistically significant differences in insulin sensitivity as assessed by the slope of glucose clearance during an ITT (Figure 3d,e,f).

### 3.4. Fto Gene Expression

**Figure 4:**
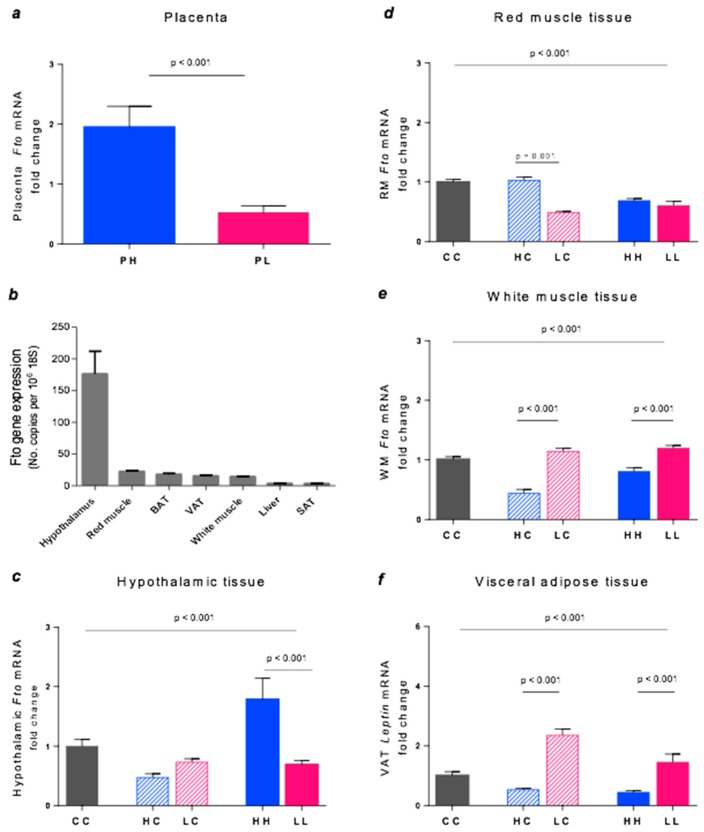
*Fto* gene expression in (**a**) placenta, (**b**) all tissues of 20-week CC mice, (**c**) hypothalamus, (**d**) RM, (**e**) WM, and (**f**) VAT. Data represent the mean ± SEM. (**a**) PH *n* = 15, PL *n* = 18. (**b**) Values were calculated for control mice (CC) and expression of *Fto* is relative to 18S. (**c**) CC *n* = 7, HC *n* = 6, LC *n* = 10, HH *n* = 5, LL *n* = 6. (**d**) CC *n* = 7, HC *n* = 7, LC *n* = 6, HH *n* = 6, LL *n* = 6. (**e**) CC *n* = 5, HC *n* = 5, LC *n* = 4, HH *n* = 5, LL *n* = 5. (**f**) CC *n* = 5, HC *n* = 7, LC *n* = 6, HH *n* = 7, LL *n* = 4.

Expression of the *Fto* gene varied significantly among the different tissues and also between treatment groups. In the placentas recovered from the high GI (PH) and low GI (PL) mothers at 18-19 days gestation, *Fto* expression was 3.8-fold higher in the high GI mothers compared to the low GI mothers (*p* = 0.0001, Figure 4a). In the offspring at 20 weeks of age, there were significant differences in *Fto* expression according to tissue type and dietary exposure. Expression of *Fto* was eight-fold higher in the hypothalamus than in the next highest tissue, red muscle (Figure 4b). Hypothalamic *Fto* gene expression was 2.5-fold higher in the HH group vs. LL (*p* = 0.0001), whereas HC and LC were similar (*p* = 0.1, Figure 4c). In red muscle, the HC group was two-fold higher than LC (*p* = 0.0001) but HH was similar to LL (Figure 4d). By contrast, the findings were the opposite in WM. *Fto* mRNA expression was almost 3-fold higher in the LC group vs. HC (*p* = 0.0001) and 1.5-fold higher in LL group vs. HH (*p* = 0.0001, Figure 4e). In VAT, control mice displayed the highest expression of *Fto* (CC vs. HH and LL, both *p* = 0.001) but there were no differences between HC and LC or between HH and LL, Figure 4f).

### 3.5. Leptin Gene Expression

**Figure 5:**
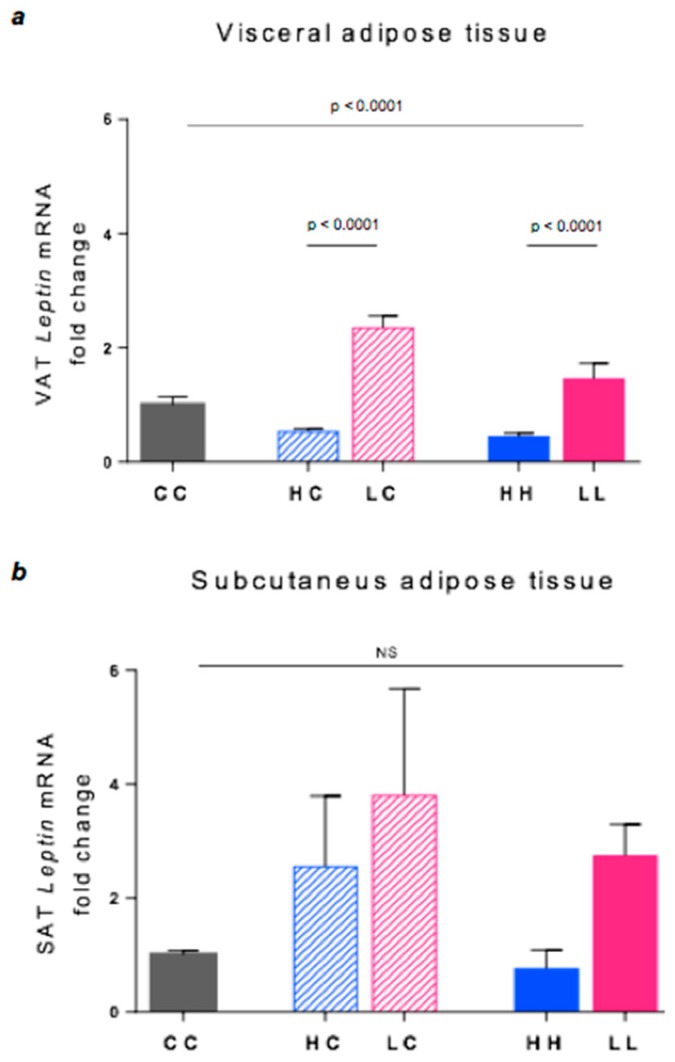
Leptin gene expression in the (**a)** VAT and (**b**) SAT. Data represent the Mean ± SEM. (**a**) CC *n* = 5, HC *n* = 6, LC *n* = 6, HH *n* = 6, LL *n* = 6. (**b**) CC *n* = 6, HC *n* = 7, LC *n* = 7, HH *n* = 4, LL *n* = 4. NS = not significant.

Marked differences in leptin expression occurred in VAT. The LC group showed 4.4-fold higher expression than HC (*p* = 0.0001), and LL was 3.3-fold higher than HH (*p* = 0.0001, Figure 5a). Similarly, in SAT, LL showed an ~3.5-fold higher leptin expression than HH, but this difference was only marginally significant (*p* = 0.02, Figure 5b).

### 3.6. Hypothalamic Appetite Gene Expression

**Figure 6:**
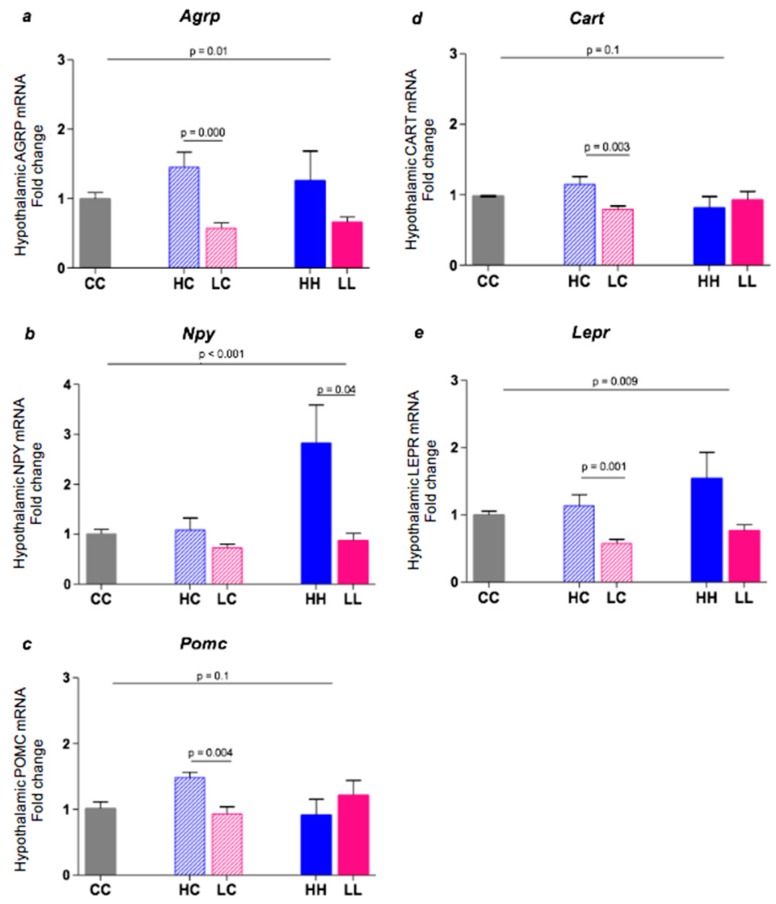
Hypothalamic *Agrp*, *Npy*, *Pomc*, *Cart*, and *Lepr* gene expression in 20-week male C57BL/6 mice. Data represent the Mean ± SEM. (**a**): CC *n* = 11, HC *n* = 7, LC *n* = 10, HH *n* = 7, LL *n* = 7. (**b**): CC *n* = 11, HC *n* = 7, LC *n* = 11, HH *n* = 9, LL *n* = 7. (**c**): CC *n* = 11, HC *n* = 6, LC *n* = 11, HH *n* = 9, LL *n* = 7. (**d**): CC *n* = 6, HC *n* = 7, LC *n* = 11, HH *n* = 9, LL *n* = 7. (**e**): CC *n* = 11, HC *n* = 7, LC *n* = 11, HH *n* = 9, LL *n* = 7.

In the hypothalamus, high GI diet offspring expressed significantly more orexigenic (appetite-stimulating) genes than low GI offspring (Figure 6). Specifically, *Agrp* mRNA expression was approximately 2.5-fold higher in HC vs. LC (*p* = 0.0001, Figure 6a), with a similar trend at 20 weeks (HH vs. CC, *p* = 0.2). *Npy* mRNA expression was 3.1-fold higher in HH vs. LL (*p* = 0.04, Figure 6b), although HC and LC were comparable to each other and the control, CC (*p* = 0.1).

Paradoxically, *Pomc* mRNA expression, a reflection of appetite inhibition, was also significantly higher in HC vs. LC (*p* = 0.004, Figure 6c), while HH, LL, and CC were similar. Likewise, *Cart* mRNA gene expression was higher in HC vs. LC (*p* = 0.003, Figure 6d), while HH, LL, and CC groups were similar. Finally, *Lepr* mRNA expression was upregulated in both HH and HC groups compared to CC (*p* = 0.001). The difference between HC and LC was significant, but not between HH vs. LL (Figure 6e).

### 3.7. Plasma Hormones

**Figure 7:**
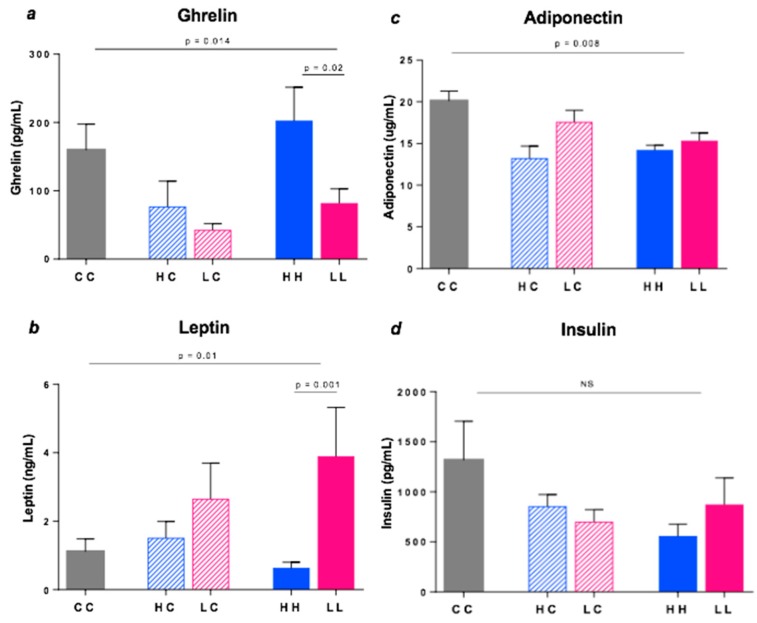
Plasma hormone levels in 20 week old mice (**a**) ghrelin, (**b**) leptin, (**c**) adiponectin and (**d**) insulin. Data represent the Mean ± SEM. CC *n* = 6, HC *n* = 8, HH *n* = 8, LC *n* = 8, LL *n* = 8. NS = not significant.

Plasma ghrelin levels varied markedly among the diet groups (Figure 7a). Specifically, ghrelin concentration was 2.5-fold higher in HH compared to LL (*p* = 0.02), with a similar trend in HC vs. LC, implying greater perceived hunger in high GI offspring. In contrast, leptin levels in plasma were over 6-fold higher in LL vs. HH (*p* = 0.001), implying greater satiety (Figure 7b). Adiponectin and insulin levels were highest in the control group (CC) but similar among the intervention groups (Figure 7c,d).

## 4. Discussion

In this study, we show that *Fto* expression is almost 4-fold higher in placenta of female mice fed a high GI vs. low GI diet during pregnancy, and >2-fold higher in the hypothalamus of offspring who continued the high GI diet from weaning to 20 weeks of age (HH vs. LL). By contrast, *Lep* expression in offspring visceral adipose tissue was >3-fold higher in the offspring of mothers consuming the low GI diet throughout pregnancy and lactation (LC vs. HC). Moreover, *Lep* expression remained >3-fold higher in offspring aged 20 weeks (LL vs. HH). Importantly, these differences occurred in the absence of differences in offspring weight, glucose tolerance, or insulin sensitivity, suggesting the effects were primary, rather than secondary to phenotypic differences. The findings imply that consumption of rapidly digestible starchy foods may increase the risk of early-onset obesity in humans. To our knowledge, our study in mice is the first to suggest that the glycaemic qualities of carbohydrate consumed during pregnancy and lactation have important effects on the programming of appetite-related genes in the offspring.

In humans, both *FTO* and *LEP* genes have been linked to BMI and regulation of food intake and energy status [40,41]. FTO was the first locus unequivocally associated by genome-wide studies with the risk of being overweight or obese, although its function was unknown at the time. It is now known that the murine *Fto* is a 2-oxyglutarate demethylase that catalyses DNA demethylation, with particularly high levels of expression in the hypothalamus, i.e., the key site for regulation of appetite in animals [42]. In mice, *Fto* overexpression results in a clear gene-dose-dependent increase in food intake and body fat, suggesting that FTO gene expression may have similar effects in humans [42]. Outside the brain, FTO risk alleles can influence the methylation of ghrelin mRNA [43], potentially explaining why plasma ghrelin levels were 2.5-fold higher in high-GI vs. low-GI offspring (HH vs LL). Ghrelin is a powerful orexigenic signal produced by the stomach, normally peaking before the onset of a meal.

In the present study, we also found higher levels of hypothalamic expression of genes transcribing key appetite-stimulating hormones, i.e., *Agrp* (3-fold higher in HC vs. LC) and *Npy* (>3-fold higher in HH vs. LL). Paradoxically, however, expression of appetite-inhibiting genes *Pomc*, *Cart* and *Lepr* was also significantly higher in offspring exposed to high GI starch until weaning, although not at 20 weeks. In the absence of differences in weight, these findings suggest that anorexigenic neuronal responses may cancel out the effect of orexigenic responses, explaining why we saw no differences in food intake up to 16 weeks of age. It is possible that weight gain differences may have been seen if both diets had been higher in fat. However, we cannot rule out the possibility of differences appearing in later adulthood (i.e., the past 20 weeks of age in mice), when differences in GI could be associated with the variation in the rate of adult weight gain.

Although we hypothesised that expression of *Fto* would differ in pregnant mice fed high vs. low GI diets, we did not expect to also see differences in *Lep* and *Lepr,* as well as in plasma ghrelin and leptin. Leptin is a major regulator of food intake and energy homeostasis in animals [44]. It is synthesised and released in response to increased energy storage in adipose tissue. Importantly, stimulation of leptin synthesis is produced by hyperglycaemia, and potentially by meal-to-meal postprandial glycaemic spikes. In the present study, both *Lep mRNA* expression in VAT and plasma leptin levels were significantly higher in the 20-week old offspring of low GI-fed mothers (LL vs. HH). Thus, despite the lack of phenotypic differences, leptin secretion was actually higher. Paradoxically, however, *Lepr* gene expression in the hypothalamus was highest in the *high* GI-fed offspring at both weaning and 20 weeks. While leptin receptor protein is found on the surface of many organs, it is in the hypothalamus where the binding of leptin to its receptor triggers a series of chemical signals that allay hunger. Taken together, this suggests that the two effects—higher of expression of *Lep* but lower expression of *Lepr* in low GI-fed offspring—may explain the lack of difference in body weight between the groups up to 16 weeks of age.

In humans and animal models, leptin also exerts inhibitory feedback on anabolic hypothalamic peptides, such as NPY, and positive feedback on catabolic peptides, such as POMC [45]. When leptin central signalling is disrupted by genetic modifications, NPY expression increases and POMC expression decreases, putting the organism into an anabolic state, and increasing the risk of obesity [45]. However, in the present study, both *Npy* and *Pomc* mRNA gene expression were significantly upregulated in high GI-fed offspring, although this varied with the length of dietary exposure (until 20 weeks and 4 weeks of age respectively). Similarly, CART expression was ~1.5 fold higher in offspring exposed to the high GI diet until weaning (HC vs. LC), implying that this anorexic gene was upregulated, perhaps to counter the effect of strong hunger signals, such ghrelin, *Agrp and Npy*. Again, the net effect may have been to cancel out the actions of each other, with no impact on food intake or weight gain.

In a systematic review and meta-analysis of 30 studies in rodents, Campbell et al. reported that a high GI diet increased five traits, including body weight, fat mass, circulating insulin level, as well as glucose and insulin area iAUC during an oral glucose tolerance test [29]. They concluded that these effects may not be a direct result of GI *per se*, but a consequence of unintended variation in other dietary constituents, such as fibre or resistant starch. In the present study, it is also possible that an unknown amount of resistant starch played a role in offspring fed the two diets into adulthood (LL and HH). However, it cannot have directly influenced energy consumption in those offspring where only the mothers received the two diets (HC vs. LC).

The strengths of this study include the use of an animal model which allowed us to observe the early *in utero* impact of the maternal diet on the expression of *Fto* and *Lep* genes in prenatal tissue (the placenta), juvenile and in adult tissues of the offspring, i.e., invasive studies not possible in humans. The treatment diets were identical in macronutrient and micronutrient composition apart from the source of the carbohydrate, allowing us to separate the effect of quantity of carbohydrate and other macronutrients from the quality of carbohydrate. The *in vitro* rate of starch digestion test conducted on the custom diets confirmed that the rate at which glucose from each diet became available for absorption varied according to their GI. A weakness is that we did not undertake postprandial studies or continuous glucose monitoring to confirm that meal-related glycaemia and/or the glucose peak were higher in mice fed the high GI diet. However, previous studies have confirmed differences after feeding in both mice and rats [46,47]. A further weakness is that we did not undertake body composition analysis in offspring, which may have detected differences in fat mass vs. lean mass in animals who were otherwise similar in weight.

## 5. Conclusions

Taken together, our novel findings provide insights into how differences in the rate of digestion and absorption of starchy foods and/or maternal glycaemia during pregnancy may program appetite and predispose offspring to obesity. Further studies are required to detect the mechanism behind differences in gene expression such as DNA methylation and histone formation. The knowledge that dietary carbohydrates can interact with obesity-associated genetic variants *in utero* and early life presents the opportunity for investigating more effective interventions to address obesity.

## Supplementary Materials

The following are available online at http://www.mdpi.com/2072-6643/10/10/1342/s1, Figure S1. Schematic representation of the study design. Table S1. Diet composition. Table S2. Oligonucleotide sequences.
