# Supplementary material for: Glycaemic Index of Maternal Dietary Carbohydrate Differentially Alters Fto and Lep Expression in Offspring in C57BL/6 Mice
Source: Nutrients. 2018 Sep 20;10(10):1342. doi: 10.3390/nu10101342 (PMC6213875; doi:10.3390/nu10101342)
Supplement: Supplementary file 1 [file nutrients-10-01342-s001.pdf]

nutrients

Supplementary information

Glycaemic index of maternal dietary carbohydrate differentially alters *Fto* and *Lep* expression in offspring in C57BL/6 mice

Theodora Sideratou[1], Fiona Atkinson[2], Grace J. Campbell[2], Peter Petocz[3], Kim S. Bell-Anderson[2,*] and Jennie Brand-Miller[2]

[1]School of Life and Environmental Sciences, University of Sydney, NSW 2006 Australia; dorasider@hotmail.com,
[2] School of Life and Environmental Sciences and Charles Perkins Centre, University of Sydney, NSW 2006 Australia; fiona.atkinson@sydney.edu.au, grace.campbell@sydney.edu.au, kim.bell-anderson@sydney.edu.au, jennie.brandmiller@sydney.edu.au
[3]Department of Statistics, Macquarie University, NSW, 2109 Australia; peter.petocz@mq.edu.au
*Correspondence: kim.bell-anderson@sydney.edu.au; Tel.: +61 2 93516267

Table S1. A comparison of the main nutrients in the two special feeds and the chow diet.

	LOW GI	HIGH GI	CHOW
Protein (g/100 g)	19.4	19.4	19.6
Total Fat (g/100 g)	7.0	7.0	4.6
Crude fibre (g/100 g)	4.7	4.7	4.5
AD fibre (g/100 g)	4.7	4.7	Not specified
Digestible energy (MJ/kg)	16.3	16.3	14.3
Total calculated digestible energy from lipids (g/100 g)	16.0	16.0	Not specified
Total calculated digestible energy from protein (g/100 g)	21.0	21.0	Not specified

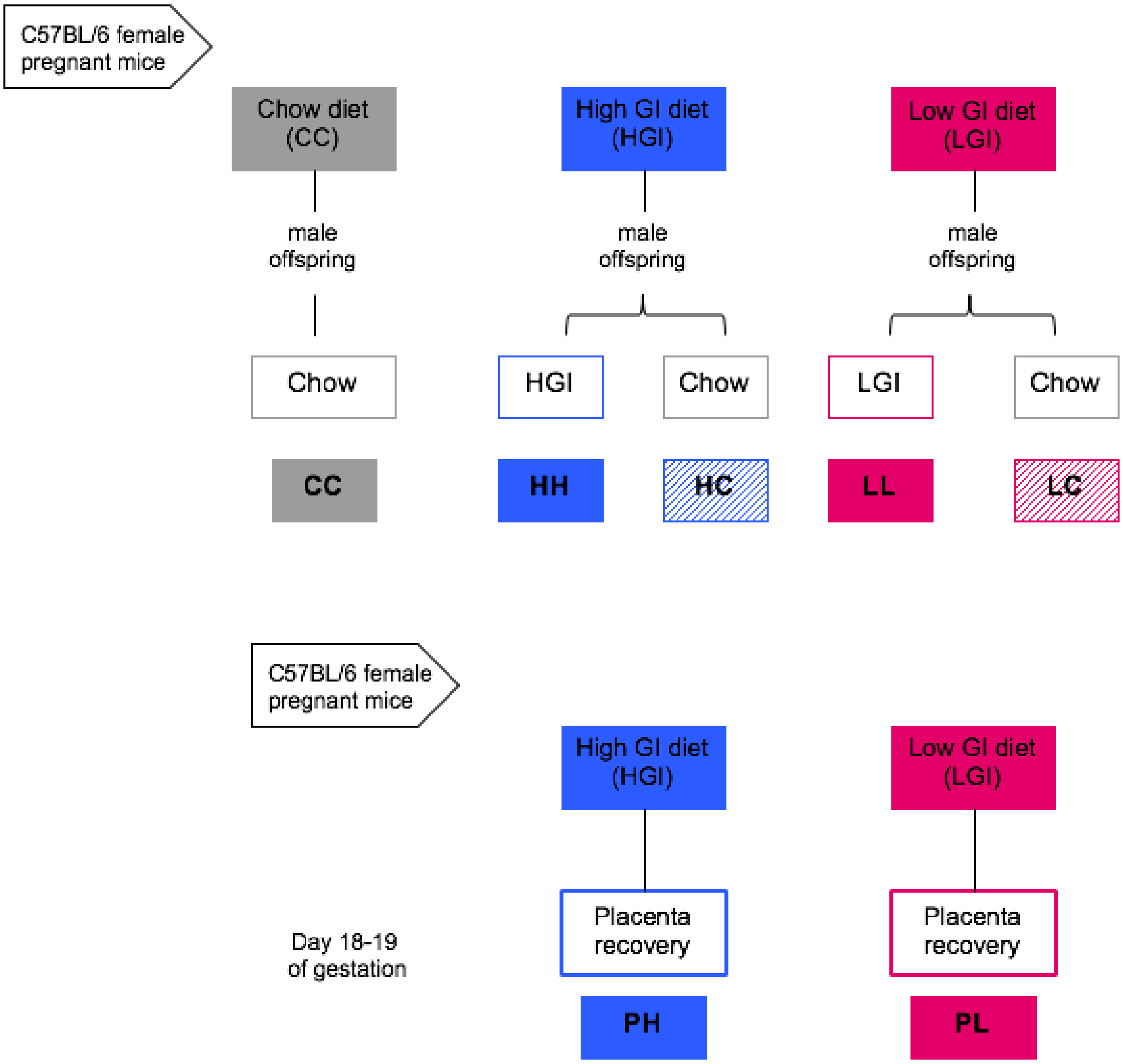

Figure S1. Schematic representation of the study design. GI: glycaemic index, CC: chow maternal, chow offspring diet, HH: high GI maternal, high GI offspring diet, LL: low GI maternal, low GI offspring diet, HC: high GI maternal, chow offspring diet, LC: low GI maternal, low GI chow diet, PH: placenta from high GI mothers, LH: placenta from low GI mothers.

Table S2: Oligonucleotide sequences.

Oligonucleotide name	Length (bp)	Tm (°C)	GC (%)	Sequence (5′- 3′)
Mus_FTO_fwd	20	64.0	60	GTG AGG ACG AGT CCA GCT TC
Mus_FTO_rev	20	64.0	55	AGC AGT CTC CCT GGT GAA GA
Mus_Lep_fwd	20	63.8	45	CTC TTT CCG GAA CAT TTG GA
Mus_Lep_rev	20	63.7	45	GCT CAG CAA TAT GCC AAC AA
Mus_Agrp_fwd	20		55	GGC CTC AAG AAG ACA ACT GC
Mus_Agrp_rev	20		55	GAC TCG TGC AGC CTT ACA CA
Mus_Npy_fwd	20		55	AGA GAT CCA GCC CTG AGA CA
Mus_Npy_rev	20		50	GAT GAG GGT GGA AAC TTG GA
Mus_Pomc_fwd	21		52	ACG TGG AAG ATG CCG AGA TTC
Mus_Pomc_rev	21		52	GCA CCA GCT CCA CAC ATC TAT
Mus_Cartpt_fwd	21		52	TAC TGC TAC CTT TGC TGG GTG
Mus_Cartpt_rev	21		52	TTC GAT CAG CTC CTT CTC GTG
Mus_Lepr_fwd	21		52	TCT GGA GCC TGA ACC CAT TTC
Mus_Lepr_rev	21		52	AGG GTC TGG TGT GGT CAA AAG
Mus_18s_fwd	20	67.6	60	CAC GGC CGG TAC AGT GAA AC

© 2018 by the authors. Submitted for possible open access publication under the terms and conditions of the Creative Commons Attribution (CC BY) license (http://creativecommons.org/licenses/by/4.0/).
